# Label-free impedance flow cytometry for nanotoxicity screening

**DOI:** 10.1038/s41598-019-56705-3

**Published:** 2020-01-10

**Authors:** Melanie Ostermann, Alexander Sauter, Ying Xue, Eivind Birkeland, Julia Schoelermann, Bodil Holst, Mihaela Roxana Cimpan

**Affiliations:** 1 Department of Clinical Dentistry, University of Bergen, Bergen, Norway; 2 Present Address: Royal Norwegian Naval Academy, Bergen, Norway; 3 Present Address: Institute for Biochemistry, ETH Zürich, Switzerland; 4 Present Address: BerGenBio ASA, Bergen, Norway; 5 Department of Physics and Technology, University of Bergen, Bergen, Norway

**Keywords:** Apoptosis, Medical research

## Abstract

The development of reliable and cost-efficient methods to assess the toxicity of nanomaterials (NMs) is critical for the proper identification of their impact on human health and for ensuring a safe progress of nanotechnology. In this study, we investigated the reliability and applicability of label-free impedance flow cytometry (IFC) for *in vitro* nanotoxicity screening, which avoids time-consuming labelling steps and minimizes possible NM-induced interferences. U937 human lymphoma cells were exposed for 24 h to eight different nanomaterials at five concentrations (2, 10, 20, 50, and 100 μg/mL). The NMs’ effect on viability was measured using IFC and the results were compared to those obtained by trypan blue (TB) dye exclusion and conventional flow cytometry (FC). To discriminate viable from necrotic cells, the IFC measurement settings regarding signal trigger level and frequency, as well as the buffer composition, were optimised. A clear discrimination between viable and necrotic cells was obtained at 6 MHz in a sucrose-based measurement buffer. Nanomaterial-induced interferences were not detected for IFC. The IFC and TB assay results were in accordance for all NMs. The IFC was found to be robust, reliable and less prone to interferences due to the advantage of being label-free.

## Introduction

Nanotechnology has gained considerable impact in a variety of areas, such as medicine, biotechnology, biomaterials, electronics, food, agriculture, energy, and consumer products, where nanomaterials (NMs) are increasingly used owing to their favourable physicochemical properties^1–3^. The widespread production and use has led to an increased exposure to NMs and raises concerns with regard to their potential adverse effects on human health and the environment^2,4,5^. To address these issues a new branch of toxicology, named nanotoxicology, has emerged^6^. A major challenge in nanotoxicity testing is the heterogeneity of NMs and the lack of standardized techniques for assessing their physicochemical properties and biological effects^3,7^. Many colorimetric *in vitro* assays, while well-established for chemicals and pharmaceuticals, have been shown to generate false-positive or false-negative results due to NM-caused interferences^5,8,9^. Kroll *et al*. (2012) found that these interferences are particle, concentration, and assay specific, and *in vitro* assays have thus to be individually adapted and optimized^10^, which makes testing of NMs difficult, time-consuming, and expensive. Therefore, first-line interference-free screening methods are urgently needed in order to detect NM formulations that are toxic early in the development, as well as to identify the concentrations and time points at which modifications occur in order to provide clues for further investigations. Based on these primary screenings, more resource-heavy mechanistic studies can be performed, thus sparing overall labour and financial resources^11–13^.

**Figure 1:**
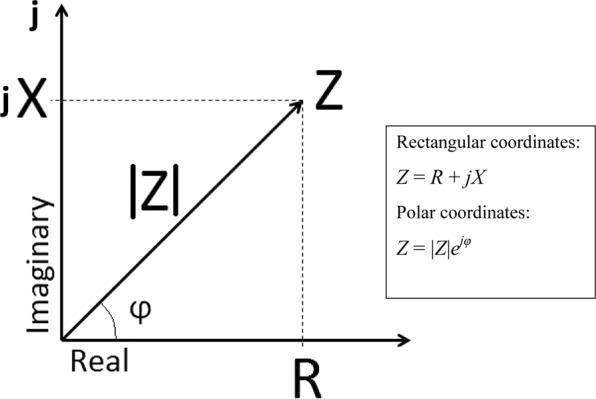
Impedance can be presented on a complex plane as the vector *Z*. When represented in rectangular coordinates, the components of the impedance *Z* are the resistance *R, w*hich is the real component, and the reactance *X*, which is the imaginary component that is dependent on frequency. When represented in polar coordinates, the components of the impedance *Z* are the magnitude *|Z|*, which is the length of the vector *Z*, and the phase *φ*, which is the angle of the vector *Z* with respect to the real axis.

Conventional flow cytometry (FC) is a widely used technique, having the advantage of rapidly assessing high numbers of individual cells^14^. However, labelling of the cells is needed and interactions of the NMs with assay reagents and fluorescence have been reported^9^. In an attempt to overcome these limitations, impedance flow cytometry (IFC) uses label-free impedance-based readings for rapid and multiparametric analysis of single cells, making it a promising future candidate to assess cellular nanotoxicity^12,15^. The value measured in IFC is the electric impedance (*Z*) of biological cells, which is a complex number consisting of two parts: the resistance (*R*, the real part of *Z*) and the reactance (*X*, the imaginary part of *Z*), which is frequency dependent.

1 $$Z=|Z|{e}^{j\varphi }=R+jX$$

where the impedance magnitude |*Z*| represents the ratio of the voltage magnitude to the current magnitude and the phase angle *ϕ* the difference between the phase of the voltage and the phase of the current^16,17^ (Fig. 1).

**Figure 2:**
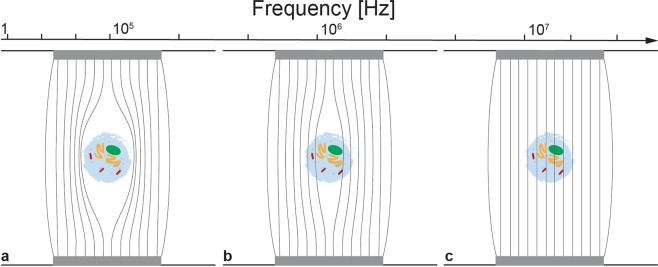
Behaviour of a cell in an electric field. (**a**) At low frequencies, the cell membrane constitutes a significant barrier to the current flow and information about the cell size is gained. (**b**) At intermediate frequencies, information of the membrane properties is revealed. (**c**) At high frequencies, the current can cross the cell membrane providing information about the cell interior. (Figure adapted and used with permission from Amphasys AG^20^).

The state of the cell can be assessed by measuring the impedance at different frequencies. The cell membrane itself acts as an insulator at low frequencies, allowing the determination of cell size^15^. At such frequencies, the cell membrane is polarized and hinders the flow of the electrical current (Fig. 2a). The charge distribution of the charged molecules on the inside of the cell changes when an oscillating electric field is applied to the cell. When medium frequencies are used, the membrane polarization decreases and the cell membrane integrity can be assessed^15^. Currents of relatively high frequency, effectively pass the membrane and thus the intracellular properties can be measured (Fig. 2b,c).

**Figure 3:**
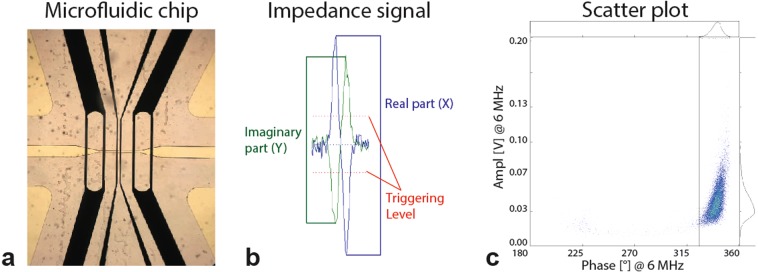
Microfluidic chip used in the IFC. Basic principles on detection of impedance signals with a typical result presented as a scatter plot. (**a**) Microfluidic chip containing two pairs of microelectrodes (two black lines in the middle of the picture). The channel size (clear horizontal stripe in the middle) can be chosen to adjust the sensitivity according to different cell sizes. (**b**) The impedance signal consists of the imaginary part (Y, green) and real part (X, blue) plotted in separate lines. The trigger level (red) sets the threshold for accepted cells/particles. (**c**) Density scatter-plots reveal electronically different cell populations. (Figure in parts adapted and used with permission from Amphasys AG)^20^.

One of the first instruments to use electric-based cell analysis was the Coulter counter, introduced by Wallace H. Coulter in the 1950s^18^, that relied on direct current or low frequency measurements^15^. Its major use was to count and determine the size of cells. In 1987 the CASY technology, also relying on electric field-based cell analysis, was introduced (Schärfe System GmbH), which besides cell concentrations also assessed cell viability based on the plasma membrane integrity. Both techniques use electrolyte-containing measurement reservoirs and impedance changes result from the volume displacement of cells/particles when passing through the apertures. Later, a microfluidic chip for cell and particle size characterization (100 samples/s) was proposed by Gawad *et al*. (2001)^19^, which combined spectral impedance, flow cytometry and the Coulter particle counter principle. This was further developed by Amphasys AG (Switzerland) and is now commercially available as a portable IFC, which employs a microfluidic chip containing two pairs of microelectrodes on each side of a microchannel (Fig. 3a). One of these pairs is used for sensing changes in the electric current at a given voltage, effectively measuring the change in impedance caused by a cell passing by, whereas the other pair acts as reference. An example of the measured impedance signal is shown in Fig. 3b and Eq. (1). It consists of an imaginary (Y, green) and real (X, blue) part. The trigger level (red) is used to set the threshold for the measurements and determines which signal is accepted as particle/cell (signal above triggering level) and what is considered noise or cell debris (signal smaller than triggering level). The measurement output is presented in form of a density scatter plot as shown in Fig. 3c, with a specific amplitude and phase revealing different cell populations.

The cell transport through the chip is achieved by pumping a suspension-buffer containing the cells. The IFC instrument (Ampha Z30) used in this study measures the electrical impedance simultaneously at up to four freely-chosen frequencies between 0.1 and 30 MHz for each cell that is passing through the electric field^20^. Either in the form of resistance and reactance or in the polar form of amplitude and phase as shown in Eq. (1), these multiparametric impedance measurements at each frequency reveal information about the cell, such as cell size, membrane properties, and intracellular density^15,20^. The information can then be related to biological key parameters like cell viability and membrane permeability^15,17^, and subsequently be used as a measure of toxicity of the tested material.

To our knowledge, IFC differs from existing commercial instruments in that it measures the electrical properties of single cells in high throughput using a wide range of frequencies. One cell can be investigated simultaneously by several frequencies, allowing valuable insight into the state of the cells based on their size, membrane, and intracellular properties. The fact that it is label-free means that the cells are not modified by chemical reagents or by extensive handling before testing. Importantly, in the case of nanotoxicity testing, this also diminishes considerably the chances for NM-induced interferences.

In the current study, we evaluated the suitability of IFC for *in vitro* nanotoxicity testing in terms of a first-line screen approach and with respect to potential NM-caused interferences. Furthermore, we optimized and standardized the IFC assay using U937 human lymphoma cells exposed to eight Organisation for Economic Co-operation and Development (OECD) reference NMs: titanium dioxide (TiO_2_) (NM-100 and NM-101), silicon dioxide (SiO_2_) (NM-200 and NM-203), zinc oxide (ZnO) (NM-110 and NM-111), and silver (Ag) (NM-300K and NM-302). The reliability of IFC was compared to that of two traditional assays: Trypan Blue (TB) dye exclusion assay and FC.

The novelty of this study is to demonstrate the IFC applicability for first-line nanotoxicity testing with apparently no measurable interferences, even for potentially ion-forming metal-based NMs.

## Results

### Dispersion quality and physicochemical characterization

Dynamic light scattering (DLS), transmission electron microscopy (TEM), and volumetric centrifugation were used to assess size distributions and zeta potentials, primary particle sizes, and effective densities, respectively (Supplementary Table S1). Detailed descriptions of the used OECD reference NMs can be found in recent JRC reports^21–24^ and in Farcal *et al*. (2015)^13^. Individual stock dispersions of the same NM showed high reproducibility between repeats (Supplementary Fig. S1). The only exception was NM-302 (Ag-rods), for which considerable sedimentation was observed within less than 20 minutes (visually detectable). Complementary information on primary particle size was collected via TEM (Supplementary Fig. S2), which revealed that the used NMs were heterogeneous, both in size and shape. Overall, the particle sizes were within the size ranges provided by the manufacturer (Supplementary Table S1 and Supplementary Table S2). Most particles exhibited a negative zeta potential between −12 and −39 mV in BSA-water, which changed to similarly negative values for all NMs once dispersed in complete cell culture medium (−6.3 to −10.3 mV). The effective densities decreased 2–3 times for all NMs compared to their raw material densities, except Ag particles, which had an approximately six times lower effective density.

### IFC optimization

**Figure 4:**
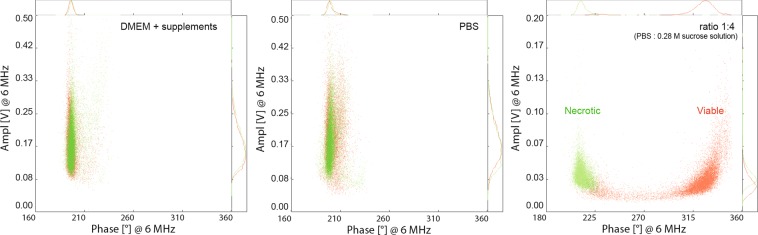
IFC measurements of U937 cells at 6 MHz using different measurement buffers. Viable (red) and necrotic cells (green) could not be separated when complete cell culture medium or PBS were used. A clear discrimination was seen when cells were suspended in a 1:4 measurement buffer (PBS: 0.28 M sucrose solution).

The main aim of this study was to assess the suitability and reliability of IFC as a first-line screening approach to evaluate the potential adverse effects of NMs *in vitro*. For this, a good understanding of the method is crucial. We optimized the measurement settings, such as the trigger level, the frequency, the gain settings, adjusted the buffer composition (high versus low buffer conductivity), and investigated whether NMs interfered with the IFC measurements. No impedance signals were detected for NMs at 100 µg/ml in measuring buffer, the only recorded signals came from the calibration beads. One key parameter to acquire reliable data is the measurement buffer, which can be adjusted to increase the contrast of damaged versus intact cell membrane for a given measurement frequency (Fig. 4).

**Figure 5:**
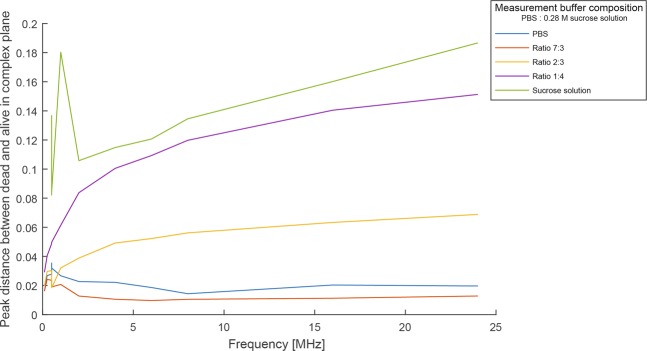
IFC measurements of U937 cells at different frequencies using various PBS: Sucrose solution ratios. Minimal differences between necrotic and viable cells were obtained at a ratio of 7:3. A clear separation between the two cell populations was found at ratios of 2:3 and 1:4 (PBS: 0.28 M sucrose solution).

Impedance measurements were carried out at different buffer conductivities and frequencies to determine the optimal sucrose concentration for separating viable from necrotic cells. The imaginary and the real part of the impedance of the cells in these measurements were plotted to obtain the individual peak distances between viable and necrotic cells in the complex plane. A summary is presented in Fig. 5. Pure PBS and a measurement buffer comprising of a 7:3 ratio of PBS and 0.28 M sucrose solution showed only minor differences between necrotic and viable cells regardless of the frequency used, with mainly overlapping populations, as shown in Fig. 5. An improvement was seen when the sucrose concentration was increased. A clear discrimination between the different cell populations (viable versus necrotic) was obtained using a buffer ratio of 2:3 and 1:4 (PBS: 0.28 M sucrose solution). The highest peak distance between necrotic and viable cells was achieved with pure sucrose solution, but artefacts appeared at low frequencies (Fig. 5).

**Figure 6:**
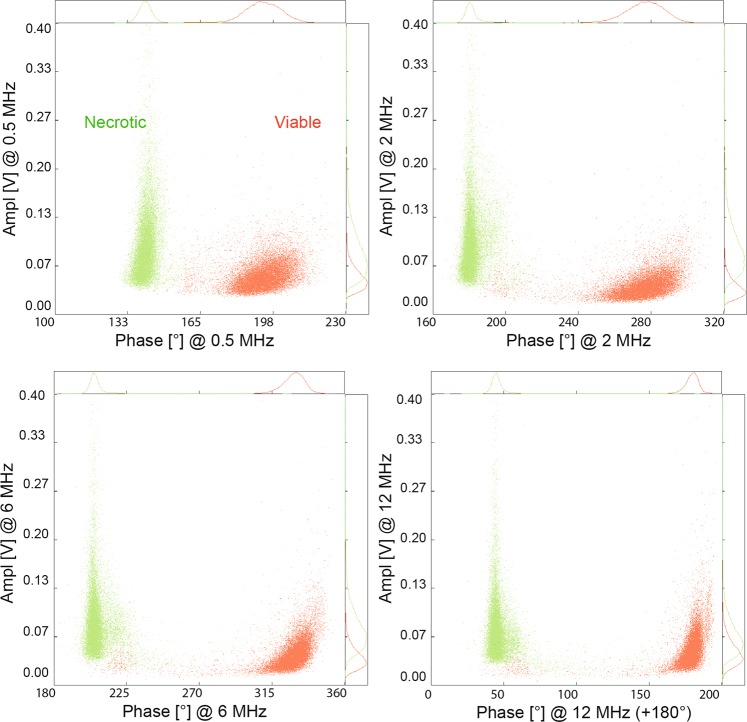
Influence of the measurement frequency on the discrimination of viable and necrotic cells. A clear separation between necrotic (green) and viable (red) cells was possible at all used frequencies using a measuring buffer ratio of 1:4 (PBS: 0.28 M sucrose solution).

**Figure 7:**
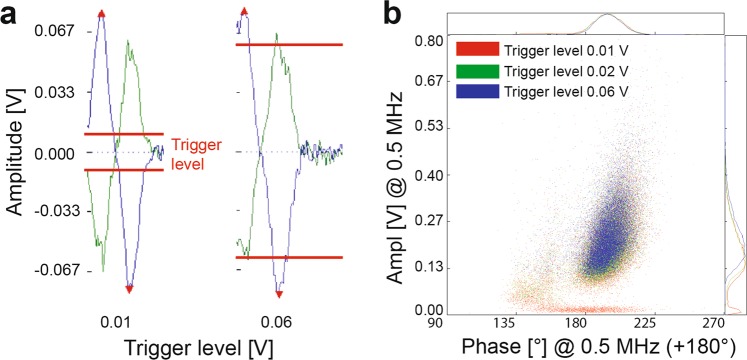
IFC measurements using different trigger levels. (**a**) The impedance signal at low trigger levels (0.01 V) is higher than the set threshold and the signal will be considered as a cell/particle, but can include measurement signals from the cell debris and electrical noise. Higher trigger levels (0.06 V) will exclude erroneous background signals, however smaller cells/particles might be lost due to the high threshold. (**b**) Density scatter plot using trigger levels of 0.01, 0.02, and 0.06 V. A low trigger level (0.01 V, red) includes signal from debris as seen as distinct population at low amplitude. This is avoided with higher trigger levels such as 0.02 V and 0.06 V. However, too high trigger levels, here > 0.06 V, might exclude cells/particles.

Apart from the buffer composition (main parameter influencing cell separation), the measurement frequency can affect the position of different cell populations in the complex plane (Figs. 6 and 7). A clear distinction, without any overlap of necrotic or viable cell populations could be achieved with any of the chosen frequencies using a low conductive buffer (ratio 1:4 PBS: sucrose solution) (Fig. 6). For further comparison of the viability data obtained by IFC and TB, we focused on the 6 MHz-frequency as according to theory, this should give information on membrane integrity.

Besides the frequency and buffer composition, also the trigger level setting has a strong impact on the measurement. The trigger level determines which cell or particle impedance would be considered of interest, as shown in Fig. 7. However, care should be taken since too low trigger levels may include erroneous background signals such as from electrical noise or cell debris (Fig. 7b).

**Figure 8:**
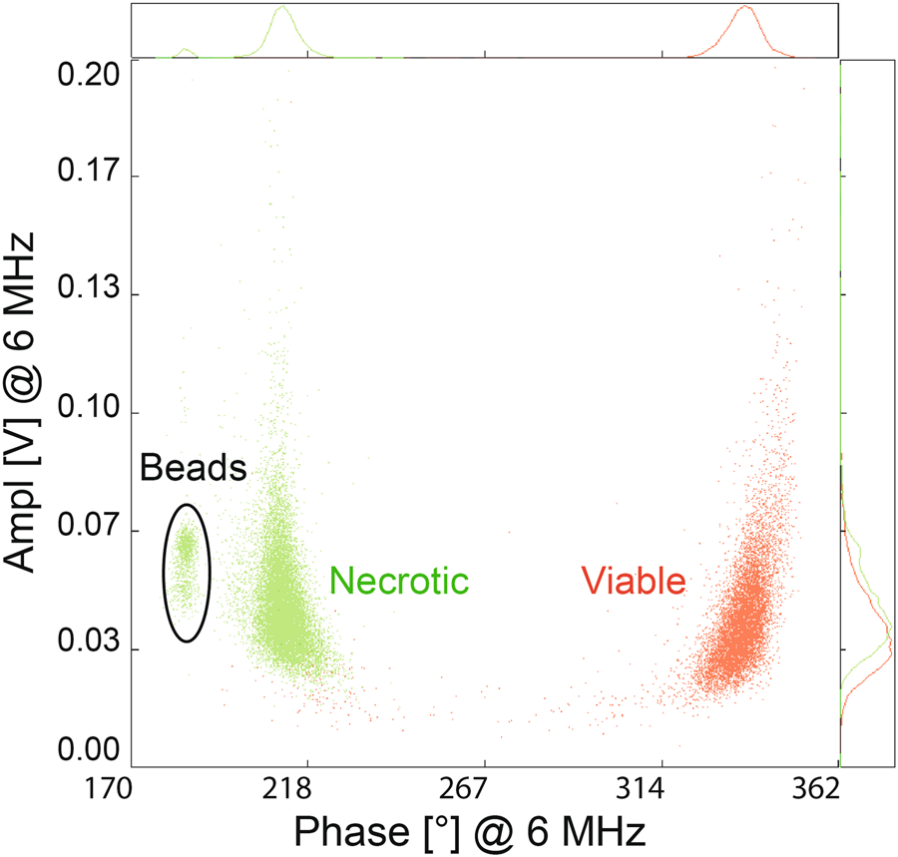
Separation of cells/particles using IFC. A clear discrimination between viable, necrotic, and smaller-sized calibration beads is possible in a 1:4 measurement buffer at 6 MHz.

The IFC can also be used to detect different-sized cells/particle sizes. Adding 10 μm polystyrene beads (Fig. 8) clearly shows the capability of the IFC to separate populations of different size or conductivity. Smaller-sized beads appeared at a lower phase angle compared to necrotic and viable U937 cells.

**Figure 9:**
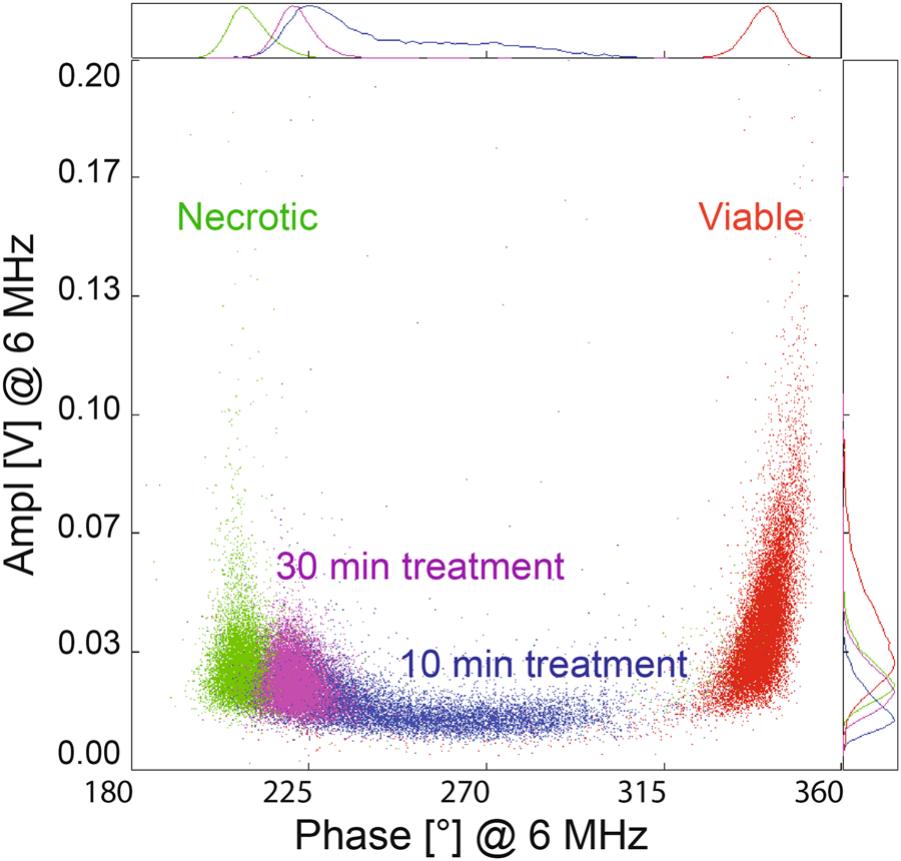
NM-300K acute effect measured by IFC. The highest concentration of NM-300K (spherical Ag, 100 μg/mL) added for 10 and 30 min before the measurement induced acute toxicity in U937 cells. An overlay of four separate measurements is shown: viable cells (red), 10 min NM-300K exposure (blue), 30 min NM-300K exposure (purple), and necrotic cells (green).

A major drawback of conventional viability assays is the often-encountered interference of NMs with reagents and/or optical readouts. In order to investigate possible NM interferences with the IFC, we also added the highest concentration of particles (100 μg/mL) right before the measurement to the positive and the negative control cells. None of the NMs were detected by the IFC and their addition to the controls did not change the viability results significantly. Only for NM-300K (nano-sized Ag) we noted an acute impact of NM-300K (nano-sized Ag) on U937 cells. Their viability was considerably reduced after 10–30 minutes exposure to NM-300 K (Fig. 9). This effect can be accounted to the release of ions from NM-300K, since interferences of the NM with the IFC should have resulted in events at the same phase angle and amplitude and should not change over time. Cells were washed in PBS prior to their resuspension in measuring buffer and therefore interferences from ions cannot explain the results. Moreover, we have checked for impedance signals from NMs in the buffer used for the measurements (PBS and isoosmotic sucrose 1:4) and no impedance signals were detected. The only signals that were detected were from the calibration beads.

### Method reliability

**Figure 10:**
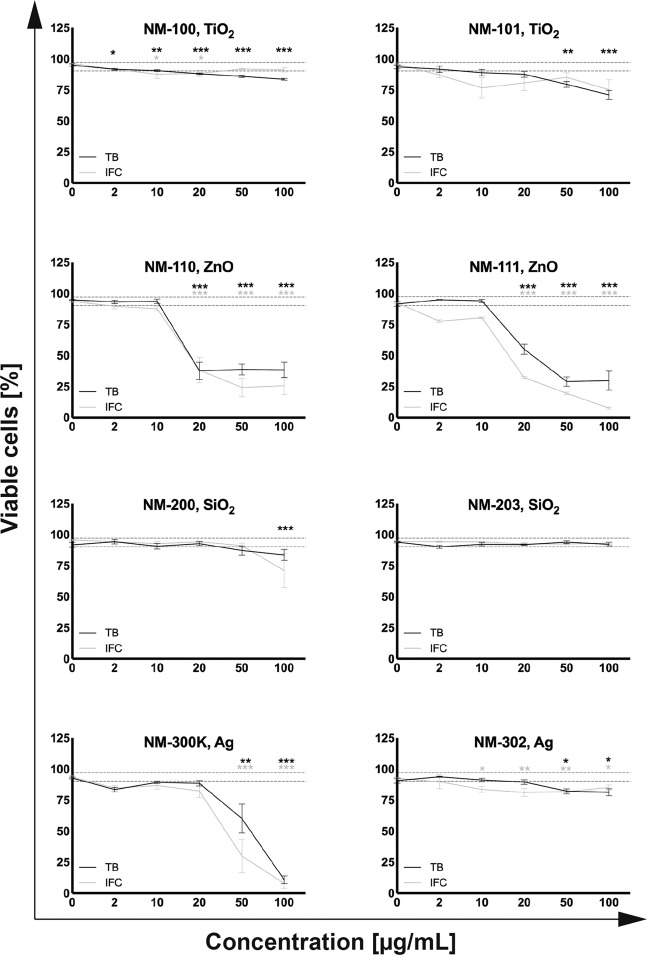
Dose-response curves: TB and IFC assays. The dotted grey line represents the mean ± SE of viable cells for all negative controls (TB and IFC) in percentages of the total count. We defined this as the “healthy cells” area. A toxic effect was judged statistically significant in comparison to the control (0 μg/mL) based on a one-way ANOVA test (*P ≤ 0.05, **P ≤ 0.01, and ***P ≤ 0.001) for n ≥ 3 individual repeats in duplicate.

U937 cells were exposed to eight OECD reference nanomaterials and viability data from a well-established viability assay (TB dye exclusion) and from FC were compared to the results obtained with the IFC. The TB assay and FC with Annexin-V and 7-AAD were chosen because they also assess the membrane integrity in the same way as IFC does in the used configuration. No interferences of the NMs with the IFC and TB assay were seen for the selected particles and U937 cells. Corresponding dose-response curves of both assays showed similar trends for all NMs at all tested concentrations (Fig. 10). A one-way ANOVA test was used to assess toxic effects (*P* ≤ 0.05) in comparison to the control (0 μg/mL).

Further analysis of the IFC and TB viability data was performed for the toxic ZnO and Ag NMs in order to investigate the accordance of the IFC results with the ones from the classical TB assay. An adaptation of the coefficient of determination (R^2^) was used to quantify this relationship (see methods section). The ZnO and Ag nanoparticles were chosen due to their non-horizontal dose-response, giving meaningful R^2^ values (Fig. 10). Non-toxic materials, on the other hand, will not result in relevant R^2^ values, since correlations of horizontal lines cannot be better than with their mean values. The R^2^ for all four compared materials was close to one, i.e., 0.978 for NM-110 (ZnO), 0.910 for NM-111 (ZnO), 0.997 for NM-302 (Ag-rods), and 0.981 for NM-300K (spherical Ag), representing a good fit, or in other words, a good correlation of the dose-response measured with these methods.

The Annexin-V/7-AAD FC assay could not be used to validate the IFC results because of NM interferences with the former, as can be seen from the representative dot plots showing viable (Annexin-V^−^ and 7-AAD^−^), apoptotic (Annexin-V^+^ and 7-AAD^−^), and necrotic (Annexin-V^+^ and 7-AAD^+^) U937 cells (Supplementary Fig. S4). Thus, the population of Annexin V^−^ cells increased in the FC readings for heated cells when adding NM-100. A significant increase of the Annexin-V^−^ and 7-AAD^−^ (viable cells) population was detected by FC when NM-100 was added 21 h prior to tumor necrosis α (TNF-α)/cycloheximide (CHX) treatment.

**Figure 11:**
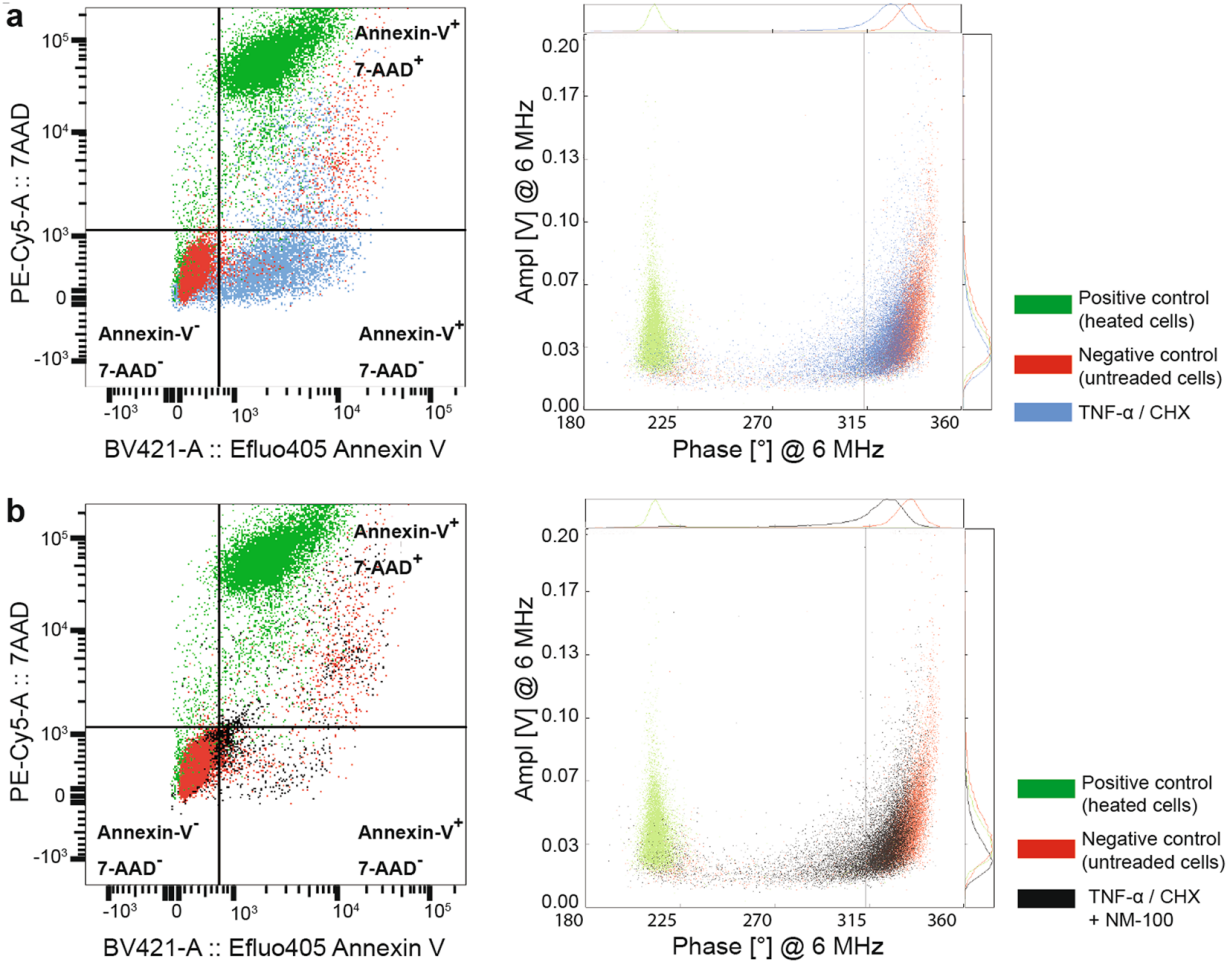
Comparison of FC and IFC measurements with regard to NM-induced interferences. Each dot plot figure represents an overlay of three individual measurements. (**a**) Negative control (unexposed cells, red), positive control (heated cells) (green), and TNF-α/CHX treated (blue) measured by FC (left) and IFC (right). (**b**) Negative control (red), positive control (heated cells) (green), and cells exposed to 100 μg of NM-100 followed by TNF-α/CHX treatment (black), measured by FC (left) and IFC (right). Adding 100 μg/mL of TiO_2_ particles (NM-100) 21 h prior to TNF-α/CHX treatment (**b**, black dots) resulted in a shift of the cell population towards the Annexin-V^−^ and 7-AAD^−^ quadrant for FC, leading to an overestimation of cell viability (left **b**). No significant changes could be seen in the IFC measurements when adding NM-100 (right **b**).

The comparison of FC and IFC results is presented in Fig. 11 where each dot plot figure represents an overlay of three individual measurements. The TNF-α/CHX treatment induced apoptosis in U937 cells, as evidenced by the presence of an Annexin-V^+^ and 7-AAD^−^ population (Fig. 11a, blue dots). This was also confirmed by TEM (Supplementary Fig. S5) and IFC. As mentioned above, when adding NM-100 21 h prior to the TNF-α/CHX treatment (Fig. 11b, black dots), the percentage of Annexin-V^−^ and 7-AAD^−^ (viable cells) was significantly higher than for cells treated only with TNF-α/CHX. This indicates an interference of NM-100 with the assay. The IFC readings were not affected by the addition of NM-100 prior to TNF-α/CHX treatment (Fig. 11b, black dots; Table 1). An increase in cell viability, but to a lower extent, was also seen in FC when the NM-100 were added right before staining (Supplementary Fig. S4, TNF-α/CHX + NM-100 (fresh)).

**Table 1:** Comparison of FC and IFC viability data presented as percentage of the total cell number.

	Viability ± SE [%]	
Sample/Treatment	FC	IFC
Negative control	89.3 ± 1.6	93.6 ± 0.5
NM-100	78.1 ± 6.9	92.0 ± 1.1
TNF-α/CHX	41.9 ± 0.9	71.4 ± 7.5
TNF-α/CHX, NM-100	74.3 ± 1.6	78.5 ± 0.3
Positive control	1.4 ± 0.4	1.5 ± 0.7

## Discussion

Interference-free screening methods to investigate the potential impact of NMs are essential to overcome the let downs of traditional viability assays. The primary aim of the present study was to establish, optimise, and evaluate a label-free impedance-based *in vitro* method to investigate NM-induced toxicity. For this, a panel of eight OECD reference NMs were used.

Critical steps in obtaining meaningful results are a thorough characterization of the physicochemical properties of the NMs and a reliable preparation of the initial stock dispersion. The determination of the particle size distribution using DLS showed a high reproducibility of single stock dispersions for low aspect ratio materials. Complementary data using TEM revealed that particles were heterogeneous both in size and shape, even though, individual stock dispersions showed high reproducibility using DLS, assuring reliable and reproducible results for the tested materials and thus reproducible conditions for the comparison of IFC with TB assay and FC.

Colorimetric assays are the golden standards to assess cell viability and cytotoxicity *in vitro*. However, contradicting data in nanotoxicity are often encountered due to interferences of the NMs with *in vitro* assays, analytical techniques^9^, and different experimental designs^25^. These classical colorimetric-based assays have been reported previously by various groups to be prone to these so called nano-specific interferences^5,9,10^. Assay reagents can adsorb onto NMs, NMs can act as fluorescence quenchers or enhancers, scatter and absorb light, thus making optical-based quantification difficult^10,26,27^.

In our study, no interferences of the eight OECD reference NMs neither with the IFC nor with the TB assay were found. The calculated R^2^-values for TB and IFC for the four most cytotoxic materials were close to one, which represents a good fit. This can be explained by the fact that both viability assays revealed, in the form they were used, the permeability status of the cell membrane. Necrotic cells are associated with a disrupted membrane, making the interior of the cell accessible for both the entry of dyes, like in the TB assay, and to ion exchange, as indirectly measured by IFC. In comparison to the TB assay, additional information about intracellular density and cell size can be gained with the IFC, when measured at higher and lower frequencies, respectively^20^. However, we focused only on intermediate frequencies (6 MHz) in our analysis, which made a straightforward method-comparison possible. Minor differences in cell viability between TB and IFC can be due to a differing sensitivity of the methods. The TB assay is an image-based labelling assay that detects later stages of cell death with cells being either stained blue (non-viable) or not stained (viable), whereas the IFC can detect earlier phases of cell death (apoptosis), which may result in slightly different viability values.

While TB and IFC results were in accordance, discrepancies were found between IFC and FC in terms of reliability. Thus, according to the FC readings, the viability of U937 cells increased as a result of adding TiO_2_ (NM-100) prior to the treatment with the apoptosis inducer cocktail TNF-α/CHX. This indicates interactions of TiO_2_ NPs with the staining agents or with the readout FC system, whereas IFC did not show any NM-specific artefacts.

The individual advantages and disadvantages of IFC as experienced by the authors are summarized in Table 2.

**Table 2:** Advantages and limitations of IFC.

Advantages	Limitations
• Label-free	• New assay: time for optimization, validation, and protocol development necessary
• Green alternative to classical assays. No toxic labelling reagents/buffers necessary	• Experience and training necessary
• Robust results: >10.000 cells/sample	• No information about the mechanism of toxicity
• Multiparametric readout possible	
• Fast	
• Less prone to nano-specific interferences	
• Various channel-sizes to adjust sensitivity	
• Increased high-throughput potential (auto sampler and barcode reader)	
• Portable equipment	

The IFC overcomes some of the limitations of traditional viability assays by measuring the electrical impedance without the need for a dye/label. The fact that the IFC does not require cell labelling, in comparison to TB and FC, simplifies sample preparation and makes it a “green” alternative for *in vitro* toxicity testing. Moreover, as evident from our results, this method may also have potential for testing of, *e.g*., chemotherapeutics and novel drug delivery tools. Similar to FC, robust results are obtained due to a high number of analysed cells (10,000 cells/sample, TB assay only 200–300 cells), including the possibility to find smaller subpopulations and different stages of cellular damage. The IFC measures each cell individually at up to four frequencies, with two parameters measured for each frequency. This multiparametric analysis can supply data about the membrane capacitance, cytoplasmic conductivity, cell density, and allows the differentiation between viable and necrotic cells^15,20^. Additionally, microfluidic chips with various channel sizes can be adapted to the size of the analysed cells in order to adjust sensitivity^28^. An auto sampler for up to 192 samples and barcode scanners are already commercially available and can be used to further automate the process and to increase the reproducibility and the throughput. Thus, the IFC shows potential as a fist-line high-throughput screening tool. However, the benefits come with an increased complexity of the system. Similar to FC, experience and training to ensure correct usage and interpretation are necessary to obtain reliable data. Moreover, the measurement buffer, trigger level and frequencies need to be fine-tuned in order to improve the signal of interest, in our case to ensure a good differentiation between necrotic and viable cells. Another crucial step is the sample preparation, where washing steps are highly recommended to minimize the amount of residual medium, which ensures a correct and reproducible buffer composition. However, this extra washing step can be a source of shortcomings, such as a loss of dead and smaller-sized cells during centrifugation. We used therefore longer centrifugation times, as usually needed in normal cell culture procedures to prevent this loss. Appropriate positive and negative controls are necessary to ensure accuracy, reliability, and precision, as well as reproducibility. Regularly rinsing and flushing of the system and chip, *e.g*., after each sample, are necessary to avoid chip and tube blockages and, more importantly, to avoid carryover of debris and leftover cells from previous samples, especially after highly toxic NMs. These cleaning procedures increase the measurement times and of the procedures in general.

As shown in this study, IFC has proven its merit for *in vitro* assessment of NM-induced toxicity. In a recent publication, our group has successfully employed the IFC to assess the toxicity of nanodiamonds used in scaffolds for bone regeneration^29^. This indicates the potential of IFC to assess the toxicity of non-metal based nanoparticles. Further work is needed to test a larger panel of NMs. The IFC is well-suited for non-adherent cells. It has also been successfully used for adherent cells^30^, however, impedance-based methods that do not necessitate trypsinization and which monitor cells *in situ*, are more suited for this type of cells. The work presented here may provide a useful starting point for the toxicity evaluation of nano-sized and larger particles using the IFC.

## Conclusion

IFC emerges as an effective, environmentally friendly, and reliable tool to determine NM-induced toxicity. This method shows a great potential to overcome essential drawbacks of classical viability assays by being label-free and thus intrinsically less prone to nano-specific interferences. The eight metal and metal oxide NMs tested having various chemical composition, size, shape, and surface coating, did not interfere with the IFC.

The IFC could be easily adapted to assess the effects of bigger particles, chemicals and pharmaceuticals. It is also more environmentally friendly than many conventional assays because it does not employ toxic or environmentally concerning agents.

The IFC stands out as a promising candidate, especially as a first-line screening tool for viability testing. We conclude that IFC is a promising method for both complementary and stand-alone testing to assess the cellular viability after NM exposure.

## Materials and Methods

### Nanomaterials

A total of eight NMs, recommended as reference materials for toxicity testing by the OECD, were chosen in order to test for possible property-specific interferences with the IFC and to investigate the reliability of the viability results. These eight reference NMs were provided by the European Commission Joint Research Centre Repository (Italy) and the Fraunhofer Institute for Molecular Biology and Applied Ecology (Germany) and were thoroughly characterized regarding relevant physicochemical properties (Supplementary Table S2). The NMs were tested for lipopolysaccharide contamination and were found to be below the admissible limit of 0.5 EU/mL (FP7 EU project NANoREG; D. Boraschi, CNR, Italy)^31^.

### Sonicator calibration and nanomaterial dispersion

The dispersion of NMs was achieved by sonication. A calorimetric calibration of a 130 W probe sonicator (VCX130, Vibra-Cell, 130 W, Sonics & Materials Inc., USA), with a 12.8 mm probe and with a regularly changed replaceable tip was used for all dispersions. A calorimetric calibration of the sonicator was adapted from the “Standard operating procedure for probe-sonicator calibration of delivered acoustic power and de-agglomeration efficiency for ecotoxicological testing, version 1.0. NANoREG”^32^. Continuous sonication for 16 min using 22% of the maximal amplitude resulted in the required acoustic energy of 7,056 Joule per vial and was thereafter used in the preparation of dispersions. Moreover, for inter-laboratory quality control, a common material, i.e, NM-200 (reference material in the FP7 EU project NANoREG) was dispersed in 0.05% w/v bovine serum albumin-water (BSA-water) to compare hydrodynamic size distributions within and between laboratories and to confirm the successful calibration of the probe. After calibration, stock dispersions of NMs (2.56 mg/mL) in BSA-water were prepared in accordance to the generic NANOGENOTOX dispersion protocol^33^. Immediately after each dispersion process, the necessary amount of NM-dispersion was added to complete cell culture medium (see section cell culture), resulting in the following exposure concentrations: 2, 10, 20, 50, and 100 μg/mL (corresponding to 1, 5, 10, 25, and 50 μg/cm^2^).

### Physicochemical characterization of nanomaterials in dispersion

The mean hydrodynamic diameter (Z-average) and zeta potential of the stock dispersions (at 25 °C) and of NM-containing cell culture medium (100 μg/mL, at 37 °C) was determined using a Zetasizer Nano ZSP (Malvern Instruments Ltd., UK)^34^. Measurements in cell culture medium were carried out prior to cell exposure (time zero) and after 24 h incubation time. The morphology of single particles was determined by TEM at 160 kV (JEM-2100, JOEL, Japan). Droplets of the stock dispersion were placed on alcian blue-treated Formvar coated copper grids using the drop-on-grid method and air-dried at room temperature^35^. The average particle size of each NM was determined using ImageJ (Version 1.50i, National Institutes of Health, USA). The effective density of NMs in cell culture medium was determined as described by DeLoid *et al*. (2014)^36^ and Cohen *et al*. (2014)^37^ using the theoretical stacking factor of 0.634 (random packing of spheres) for all NMs^38^.

### Cell culture

The human histiocytic lymphoma cell line U937 (CRL-1593.2; American Type culture Collection, Manassas, USA) was cultured in 175 cm^2^ flasks and maintained in complete cell culture medium (hereafter referred as medium) composed of Dulbecco’s Modified Eagle Medium (DMEM) (Life technologies-Gibco) and supplemented with 10% v/v Fetal Bovine Serum (FBS) (HyClone) and 1% v/v Penicillin/Streptomycin (10,000 U/mL penicillin and 10,000 U/mL streptomycin, HyClone). The suspension cells were kept in a humidified atmosphere at 37 °C, 5% CO_2_ and were subcultured every 2–3 days at a seeding density of 0.5–1 million cells/mL. Cultured cells tested negative for mycoplasma (MycoAlert PLUS detection kit, Lonza).

### Cell seeding and exposure to nanomaterials

U937 cells with a viability above 90% (tested using 0.4% TB solution) were seeded 24 h prior to NM exposure onto two identical 6-well plates at a density of 1 × 10^5^ cells/cm^2^. Next, cells were spun down within the culture dish (10 min, 160 g), the supernatant was carefully removed, and the cells were incubated for 24 h with 2, 10, 20, 50, and 100 μg/mL NM-containing medium. Untreated cells served as negative controls (0 μg/mL). Each assay was performed in duplicate on separate plates and individual experiments were repeated at least three times.

### Cell viability assessed by IFC

After NM-exposure, the cells were centrifuged (10 min, 160 g), the supernatant was removed, and the cells were resuspended in 1 mL PBS and then transferred into Eppendorf safe-lock tubes for further centrifugation (10 min, 160 g). The supernatant was carefully discarded, the cell pellets were resuspended in 100 μL PBS, and stored on ice until measured. Before each measurement, 400 μL of a 0.28 M aqueous sucrose solution (final buffer ratio 1:4 PBS: sucrose solution) was added to allow physiological osmolality, while keeping a low buffer conductivity compared to the normal intracellular conductivity of healthy cells. This provided a good contrast between healthy and dead cells as judged based on the loss of membrane integrity (Fig. 11). To ensure that cells maintained their viability in this buffer for the whole duration of the testing, the viability of the control (unexposed) cells was also measured at the end of the testing session. Cells heated at 70 °C for 30 min served as positive control for necrosis. To test for possible NM interferences, the highest NM concentration (100 μg/mL) was added to cell-free medium, negative and positive cell controls. Impedance-based measurements (Ampha Z30, Amphasys AG, Switzerland) were carried out using 0.5, 2, 6, and 12 MHz and a microfluidic chip with a channel of a 50 μm x 50 μm-sized cross section. The used settings are summarised in Table 3. The signal to noise ratio was 1:8 to 1:13 (Fig. 7). To ensure that the impedance readings did not come from the NMs, the impedance was measured for all NMs in measuring buffer and no signal was detected (see results section). The NMs and cell debris were excluded at the chosen settings, as shown in Fig. 7. The trigger level of 0.02 V would, as demonstrated in Fig. 7, exclude any noise by a good margin. Additional curve detection further excludes random noise as noise does not follow the same time-dependent pattern as cells do.

**Table 3:** Measurement settings for IFC.

Measurement settings	
Trigger level	0.02 V
Trigger frequency	0.5 MHz
Trigger source	Real part (x) with initial upward (+) deflection
Modulation (range 1–5)	3
Amplifier (range 1–8)	3
Demodulation (range 0–8)	2
Pump speed	80–100 rpm

**Figure 12:**
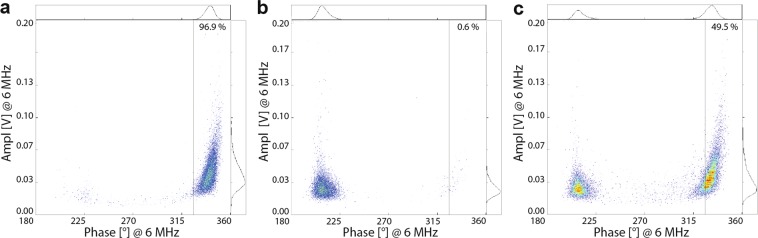
Gating for IFC measurements. (**a**) The population of viable cells was determined from the negative control and the gate was applied to all samples within one experiment. (**b**) Necrotic (heated) cells, and (**c**) 50:50 mixture of viable and necrotic cells. The cell number within the gates is expressed as percentage of the total cell count.

A total of 10,000–20,000 cells per sample were analysed using the AmphaSoft v1.2.8 and AmphaSoft 2.0 (Amphasys AG, Switzerland). The 6 MHz-frequency was found to be optimal for assessing plasma membrane permeability (Figs. 2, 5, and 6). A gate for viable cells (intact cell membrane) was created in AmphaSoft 2.0 using the negative control and then applied to all samples within one exposure experiment as shown in Fig. 12. An additional phase shift of 180° was applied to all samples when files were converted from AmphaSoft v1.2.8 to AmphaSoft 2.0 (recalibration). The viable cells within the gate were presented as a percentage of the total number of cells, which was used for further analysis.

### Trypan Blue assay

After NM-exposure, cells were collected, washed, and transferred into Eppendorf safe-lock tubes as described for the IFC assay. A 1:1 mixture, (0.4% TB solution: cell suspension) was injected into cell counting slides and the cell viability in percentage of all measured cells was determined using an automated viability cell counter (Countess, Invitrogen, USA). Untreated cells were used as negative control. In addition, medium containing the highest NM-concentration was analysed to identify possible interferences.

### Comparison of IFC and FC regarding NM-interferences

Tumour necrosis factor alpha (TNF-α) (100 Ul/mL, Sigma Aldrich) in combination with cycloheximide (CHX) (2 μg/mL, Sigma Aldrich) were used to induce apoptosis in the U937 cells. For this, cells were seeded onto two identical 6-well plates (IFC) or onto 12-well plates (FC) at a density of 2 × 10^5^ cells/cm^2^ and treated for 3 h with TNF-α/CHX. In parallel, samples treated with 100 μg/mL NM-100 (TiO_2_) for 21 h followed by a 3 h TNF-α/CHX treatment were prepared to test for NM-related interferences.

To identify apoptotic and necrotic cells by FC, the cells were labelled using an Annexin-V/7-AAD Apoptosis Detection Kit eFluor 450 (Affymetrix, eBioscience) according to the suppliers recommendations. Briefly, cells were treated with the respective agents for 24 h and then washed with PBS at 160 g for 5 min. After the cells were resuspended in 300 μl binding buffer, they were stained with 10 µl Annexin-V eFluor 450 and 5 µl 7-AAD per sample. Stained cells were immediately analysed on a BD LSR Fortessa flow cytometer (BD Biosciences, USA). A 561-nm laser was used for excitation and a 661/20 nm band pass filter was used for the detection of 7-AAD, while for Annexin-V eFluor 450 staining a 497-nm laser was used for excitation and a 450/50 nm band pass filter for reading. Viable cells from the negative control were used to set gates for live cells (7-AAD^−^ and Annexin-V^−^). 7-AAD^+^ cells were considered as necrotic and Annexin-V^+^ and 7-AAD^−^ cells were considered to be apoptotic. The FlowJo version 10 (FlowJo, LCC, USA) was used for the FC data analysis.

In parallel to FC, cells for IFC measurements were prepared, collected, and analysed as described above.

### Statistical analysis

All the data are presented as the mean ± standard error of the mean (SE) of at least three independent experiments, if not stated otherwise. An adaptation of the coefficient of determination (R^2^, Eqs. 2–4) was used as a measure of association between the cytotoxicity obtained by the TB assay and the IFC assay. Here, the mean values for each concentration of the TB assay were used as the reference and were compared to the measurements made by the IFC for each concentration. The following formulas have been used:

2 $${R}^{2}=1-\frac{S{S}_{res}}{S{S}_{tot}},$$

with

3 $$S{S}_{res}=\sum _{i}{({y}_{i}-{f}_{i})}^{2}$$

and

4 $$S{S}_{tot}=\sum _{i}{({y}_{i}-\tilde{y})}^{2},$$

with SS_res_ as the residual sum of squares, SS_tot_ being the total sum of the square, y_1_…y_n_ as the values within the IFC assay, $\tilde{y}$ being the mean of the observed data, and f_1_…f_n_ associated with the values from TB, substituting the theory in regular *R*^2^ calculations. Statistical significant differences were determined using a one-way ANOVA (*P* ≤ 0.05) with cells exposed to NM versus untreated control cells (0 μg/mL) using GraphPad Prism 6.0c (GraphPad Software, Inc., USA).

## Supplementary information

Dataset 1.

## Data Availability

All relevant data are within the paper and its Supporting Information files. The datasets used and/or analysed during the current study are available from the corresponding author on reasonable request.
