# Supplementary material for: Label-free impedance flow cytometry for nanotoxicity screening
Source: Sci Rep. 2020 Jan 10;10:142. doi: 10.1038/s41598-019-56705-3 (PMC6954202; doi:10.1038/s41598-019-56705-3)
Supplement: Supplementary file 1 — Dataset 1. [file 41598_2019_56705_MOESM1_ESM.pdf]

Supplementary Information

Label-free impedance flow cytometry for nanotoxicity screening

Melanie Ostermann[1], melanie.ostermann@uib.no
Alexander Sauter[1,2], asauter@fhs.mil.no
Ying Xue[1], ying.xue@uib.no
Eivind Birkeland[1,3], eiv.birk@gmail.com
Julia Schoelermann[1,4], julia.schoelermann@gmail.com
Bodil Holst[5], bodil.holst@uib.no
*Mihaela Roxana Cimpan[1], mihaela.cimpan@uib.no

*Correspondence: **mihaela.cimpan@uib.no**
[1]Department of Clinical Dentistry, University of Bergen, Norway.
[2]Present address: Royal Norwegian Naval Academy, Bergen, Norway.
[3]Present address: Institute for Biochemistry, ETH Zürich, Switzerland.
[4]Present address: BerGenBio ASA, Bergen, Norway.
[5]Department of Physics and Technology, University of Bergen, Norway.

Table S1: **Physicochemical properties of NMs in 0.05 % w/v BSA-water (stock solution) and complete cell culture medium (exposure medium).** Abbreviations: DLS – Dynamic Light Scattering, PDI – Polydispersity index, ELS – Electrophoretic light scattering, TEM – Transmission Electron Microscopy, $\boldsymbol{\rho}_{\mathbf{NM}}$ – Density of raw NMs, SF – Stacking Factor, and $\boldsymbol{\rho}_{\mathbf{EV}}$ – Effective density estimated by volumetric centrifugation (measurements were performed at least in duplicates / triplicates).

[a] Measurement of particle clusters.

Table S2: Overview of physicochemical properties of NMs. Abbreviations: XRD - X-ray Diffraction, EDS - Energy-dispersive X-ray spectroscopy, TEM - Transmission Electron Microscopy, BET - Brunauer–Emmett–Teller theory for specific surface area, DLS - Dynamic Light Scattering, ICP-OES - Inductively Coupled Plasma Optical Emission Spectrometry, PDI – Polydispersity index, ISP – isoelectric point, BSA – Bovine serum albumin, PBS – Phosphate-buffered saline, ppm – parts per million. Data was collected from the following references: JRC reports [21–24] and NANoREG technical data sheets [39–45].

Figure S1: Intensity-based size distributions. Individual repeats (n > 3) of NM stock dispersions in 0.05 % w/v BSA-water. Each size spectra is the average of ten individual DLS measurements ± SE conducted using automatic optimization. (**a**) NM-100 (TiO_2), (**b**) NM-101 (TiO_2), (**c**) NM-200 (SiO_2), (**d**) NM-203 (SiO_2), (**e**) NM-110 (ZnO), (**f**) NM-111 (ZnO), (**g**) NM-300K (Ag), and (**h**) NM-302 (Ag-rods).

Figure S2: Transmission electron micrographs of NMs dispersed in 0.05 % w/v BSA-water. Morphology of (**a**) NM-100 (TiO_2), (**b**) NM-101 (TiO_2), (**c**) NM-200 (SiO_2), (**d**) NM-203 (SiO_2), (**e**) NM-110 (ZnO), (**f**) NM-111 (ZnO), (**g**) NM-300K (Ag), and (**h**) NM-302 (Ag-rods) (scale bar: 200 nm).

Figure S4: Representative flow cytometry dot plots for Annexin-V eFluor450 and 7-AAD staining. To check for NM-induced interferences, 100 µg/mL NM-100 TiO_2 particles were added prior to treatment of U937 cells with TNF-α / CHX (positive control for apoptosis) and to heating cells at 70°C for 30 min (positive control for necrosis). Annexin-V eFluor 450 and 7-AAD staining were used to detect apoptotic and necrotic cells. The addition of NM-100 prior to TNF- α /CHX treated cells resulted in an overestimation of viable (Annexin V- / 7-AAD-) cells as compared to cells treated only with TNF- α / CHX. The Annexin V- population increased when NM-100 were added prior to cell heating.

Figure S5: Transmission electron micrographs of U937 cells. (a) Viable (untreated) cells and **(b)** after TNF-α / CHX treatment. Scale bar = 10 µm. (TEM image by CI Vamanu, published with permission).

Table S1 Physicochemical properties of NMs in 0.05 % w/v BSA-water (stock solution) and complete cell culture medium (exposure medium).

Sample	Mean hydrodynamic diameter ± S.E. [nm] (DLS)	PDI ± S.E.	Zeta potential ± S.E. [mV] (ELS)	Mean particle size ± S.E. [nm] (n>28) (TEM)	ρ_{NM} (g/cm^3)	SF	ρ_{EV} ± S.E. (g/cm^3)
**NM-100 (TiO2)**							
0.05 % w/v BSA-water	249.6 ± 2.2	0.169 ± 0.003	0.1 ± 0.1	141 ± 4			
(Supplier)	(230)	(0.187)					
DMEM/10% FBS, t 0 hrs	303.2 ± 7.9	0.166 ± 0.020	-9.5 ± 0.1				
DMEM/10% FBS, t 24 hrs	294.6 ± 0.7	0.148 ± 0.023	-9.5 ± 0.1		4.23	0.634	1.844 ± 0.152
**NM-101 (TiO_2)**							
0.05 % w/v BSA-water	478.6 ± 4.1	0.334 ± 0.008	-2.2 ± 0.3	71 ± 4[b]			
(Supplier)	(423)	(0.275)					
DMEM/10% FBS, t 0 hrs	522.3 ± 14.3	0.364 ± 0.015	-9.7 ± 0.2				
DMEM/10% FBS, t 24 hrs	368.7 ± 10.5	0.346 ± 0.015	-9.9 ± 0.1		4.23	0.634	1.554 ± 0.041
**NM-110 (ZnO)**							
0.05 % w/v BSA-water	233.9 ± 0.6	0.111 ± 0.002	-13.2 ± 0.3	107 ± 5			
(Supplier)	(233)	(0.110)					
DMEM/10% FBS, t 0 hrs	255.0 ± 1.8	0.123 ± 0.013	-11.7 ± 0.9				
DMEM/10% FBS, t 24 hrs	237.1 ± 4.9	0.129 ± 0.001	-10.3 ± 0.3		5.61	0.634	1.870 ±0.348
**NM-111 (ZnO)**							
0.05 % w/v BSA-water	244.3 ± 1.9	0.127 ± 0.004	-12.1 ± 0.3	80 ± 7			
(Supplier)	(247)	(0.125)					
DMEM/10% FBS, t 0 hrs	237.2 ± 1.6	0.166 ± 0.004	-10.1 ± 0.1				
DMEM/10% FBS, t 24 hrs	238.9 ± 3.4	0.141 ± 0.006	-6.3 ± 1.6		5.61	0.634	1.661 ± 0.063

**NM-200 (SiO2)**							
0.05 % w/v BSA-water	257.6 ± 8.3	0.458 ± 0.017	-33.1 ± 2.1	169 ± 21[a]			
(Supplier)	(253)	(0.387)					
DMEM/10% FBS, t 0 hrs	274.8 ± 14.8	0.466 ± 0.045	-7.9 ± 0.6				
DMEM/10% FBS, t 24 hrs	167.0 ± 0.7	0.588 ± 0.025	-8.3 ± 0.7		2.65	0.634	1.231 ± 0.011
**NM-203 (SiO_2)**							
0.05 % w/v BSA-water	144.6 ± 1.2	0.205 ± 0.010	-39.2 ± 1.4	128 ± 16[a]			
(Supplier)	(145)	(0.203)					
DMEM/10% FBS, t 0 hrs	162.5 ± 9.7	0.326 ± 0.001	-8.1 ± 0.1				
DMEM/10% FBS, t 24 hrs	N/A	N/A	-7.2 ± 0.3		2.65	0.634	1.097 ± 0.008
**NM-300K (Ag)**							
0.05 % w/v BSA-water	79.8 ± 0.2	0.260 ± 0.002	-4.5 ± 0.3	19 ± 0			
(Supplier)	(87)	(0.259)					
DMEM/10% FBS, t 0 hrs	110.6 ± 6.9	0.369 ± 0.031	-8.9 ± 0.1				
DMEM/10% FBS, t 24 hrs	108.4 ± 3.2	0.374 ± 0.017	-9.2 ± 0.6		10.49	0.634	1.889 ± 0.064
**NM-302 (Ag-rod)**							
0.05 % w/v BSA-water	878.9 ± 88.9	0.641 ± 0.040	-17.3 ± 0.7	804 ± 104			
(Supplier)	(797)	(0.527)					
DMEM/10% FBS, t 0 hrs	1032.5 ± 55.1	0.682 ± 0.017	-8.4 ± 0.9				
DMEM/10% FBS, t 24 hrs	N/A	N/A	-9.5 ± 0.2		10.49	0.634	1.966 ± 0.095

Measurements of the stock dispersion and NMs in complete cell culture medium were performed at least in triplicates and duplicates, respectively. Abbreviations: DLS – Dynamic Light Scattering, PDI – Polydispersity index, ELS – Electrophoretic light scattering, TEM – Transmission Electron Microscopy, ρ_{NM} – Density of raw NMs, SF – Stacking Factor, and ρ_{EV} – Effective density estimated by volumetric centrifugation.

[a] Measurement of particle clusters.

Table S2: Overview of physicochemical properties of NMs.

Material code	Material	Polymorph (XRD)	Chemical composition [wt%] (EDS)	Primary particle size ± SD or size range [nm] (TEM)	Morphology (TEM)	BET [m^2/g]	z-Average [nm] and PDI (DLS; 0.05% BSA-water)	Zeta potential ± SD in PBS [mV] and isoelectric point (ISP) [pH]
**NM-100**	TiO_2	Anatase	Ti 58.57, O (calculated) 40.08; traces (ppm) of Si, P, Al, K, Cr, Fe	100 ± 57	Primary sub-units are equi-axed. Aggregates/agglomerates are more fractal-like with minor spheroidal particles.	9 - 10	z-Average: 230<br>PDI : 0.187	Zeta pot: -50 ± 10<br>ISP: N/A
**NM-101**	TiO_2	Anatase	Ti 58.79, O (calculated) 40.35; traces (ppm) of Si, P, Al, S	5.5 ± 0.7	Primary particles more or less euqi-axedor slightly elongated.	234 - 316	z-Average: 449<br>PDI: 0.315	Zeta pot: -33 ± 9<br>ISP: 6
**NM-110**	ZnO	Hexagonal zincite structure	Zn 89.90, O 10.01; traces (ppm) of Si, Al, Ca, Ni, Pb, Co (ICP-OES)	Population 1= 20 - 250<br>Population 2= 50 - 350	Population 1 = hexagonal, Population 2 = cubic, tetragonal and orthorhombic.	12 -13	z-Average: 233<br>PDI: 0.110	Zeta pot: -47 ± 9<br>ISP: N/A
**NM-111**	ZnO	Hexagonal zincite structure	Zn 87.39, O 12.48; taces (ppm) of Ni, Pb, Co, Si, Al, Ca (ICP-OES)	Population 1= 20 - 200<br>Population 2= 10 - 450	Polyhedral with variable morphologies: hexagonal, cubic, tetragonal, and orthorhombic.	15-16	z-Average: 247<br>PDI: 0.125	Zeta pot: -39 ± 6<br>ISP: N/A
**NM-200**	SiO_2	Synthetic amorphous silica	Si 44.77,O 53.02; traces (ppm) of Fe, K, Mg, Zr, Al, Na, S (ICP-OES)	14 ± 7	Sub-units are equi-axed and rounded or slightly elongated (sphericity 0.39).	189	z-Average: 253<br>PDI: 0.387	Zeta pot: -48 (milliQ water, pH 7)<br>ISP: < 2
**NM-203**	SiO_2	Synthetic amorphous silica	Si 46.32, O 53.21; traces (ppm) of Al, S	13 ± 6	Sub-units are equi-axed and rounded or slightly elongated (sphericity 0.35).	204	z-Average: 145<br>PDI: 0.203	Zeta pot: -46 (milliQ water, pH 6.6)<br>ISP: < 2
**NM-300K**	Ag	Metallic	Ag 99.84, O 0.16	Majority around 20	Majority is round shaped; others are triangular or trapezium-like.	N/A	z-Average: 87<br>PDI: 0.259	Zeta pot: -11 ± 3 (ultrapure water)<br>ISP: N/A
**NM-302**	Ag	Metallic	Ag 100 (EDS)	Width: 183 ± 80<br>Length: 2700 ± 2200	Fibres, acicular.	N/A	z-Average: 665<br>PDI: 0.539	N/A

Abbreviations: XRD - X-ray Diffraction, EDS - Energy-dispersive X-ray spectroscopy, TEM - Transmission Electron Microscopy, BET - Brunauer–Emmett–Teller theory for specific surface area, DLS - Dynamic Light Scattering, ICP-OES - Inductively Coupled Plasma Optical Emission Spectrometry, PDI – Polydispersity index, ISP – isoelectric point, BSA – Bovine serum albumin, PBS – Phosphate-buffered saline, ppm – parts per million. Data was collected from the following references: JRC reports [21]–[24] and NANoREG technical data sheets [39–45]. [39] National Research Centre for the Working Environment. Technical data sheet – Material code: NM-101. http://www.nanoreg-materials.eu/Documentation/NM-101_NRG-TDS_08.05.15.pdf. [40] National Research Centre for the Working Environment. Technical data sheet – Material code: NM-110. http://www.nanoreg-materials.eu/Documentation/ NM-110_NRG-TDS_08.05.14.pdf. [41] National Research Centre for the Working Environment. Technical data sheet – Material code: NM-111. http://www.nanoreg-materials.eu/Documentation/NM-111_NRG-TDS_08.05.14.pdf. [42] National Research Centre for the Working Environment. Technical data sheet – Material code: NM-200. http://www.nanoreg-materials.eu/Documentation/NM-200_NRG-TDS_08.05.15.pdf. [43] National Research Centre for the Working Environment. Technical data sheet – Material code: NM-203. Available at: http://www.nanoreg-materials.eu/Documentation/NM-203_NRG-TDS_08.05.15.pdf. [44] National Research Centre for the Working Environment. Technical data sheet – Material code: NM-300K. http://www.nanoreg-materials.eu/Documentation/ NM-300K_NRG-TDS_02.02.15.pdf. [45] National Research Centre for the Working Environment. Technical data sheet – Material code: NM-302. http://www.nanoreg-materials.eu/Documentation/NM-302_NRG-TDS_02.02.15.pdf.

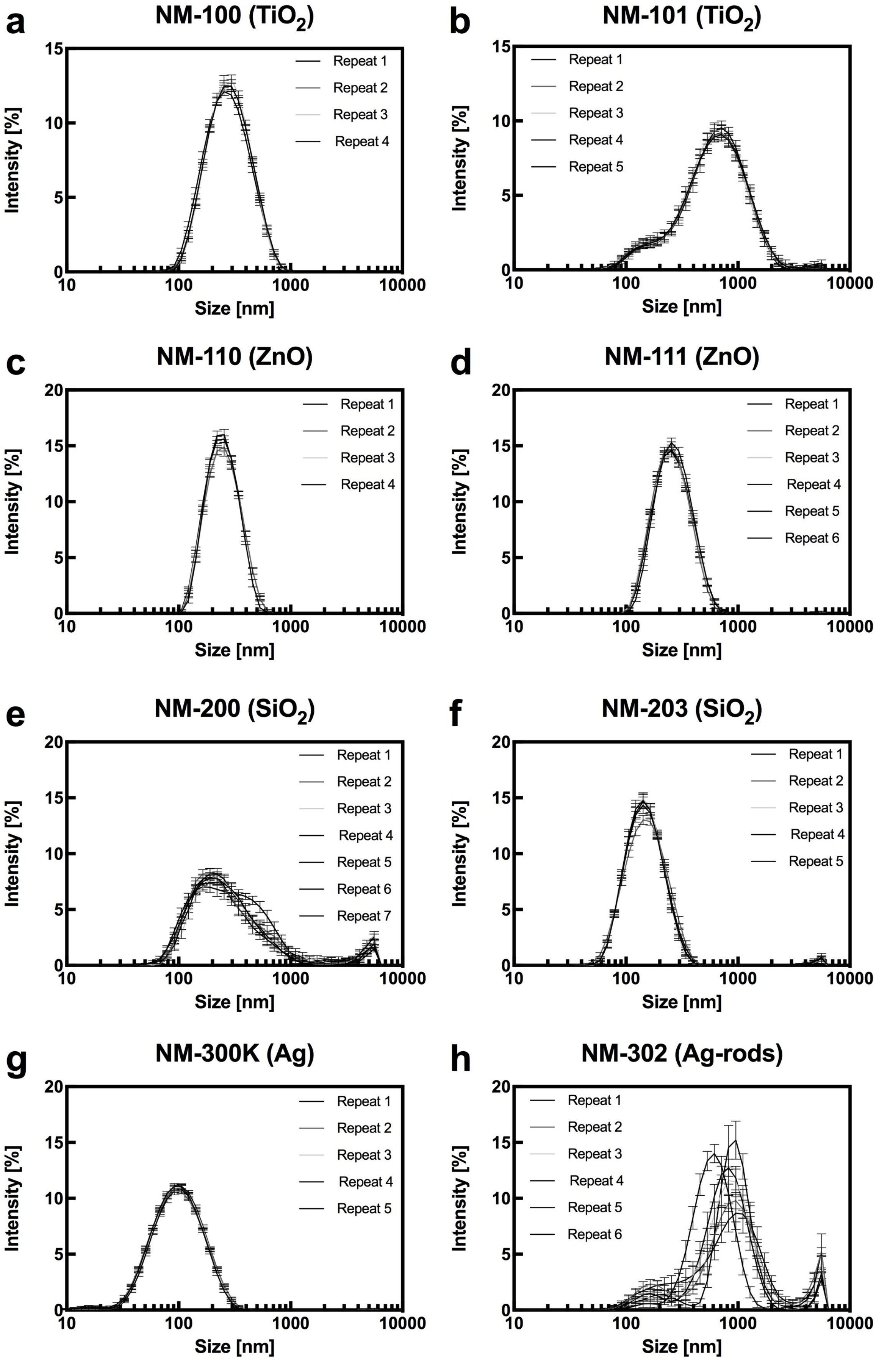

Figure S1: Intensity-based size distributions. Individual repeats (n > 3) of NM stock dispersions in 0.05 % w/v BSA-water. Each size spectra is the average of ten individual DLS measurements ± SE conducted using automatic optimization. (**a**) NM-100 (TiO2), (**b**) NM-101 (TiO2), (**c**) NM-200 (SiO2), (**d**) NM-203 (SiO2), (**e**) NM-110 (ZnO), (**f**) NM-111 (ZnO), (**g**) NM-300K (Ag), and (**h**) NM-302 (Ag-rods).

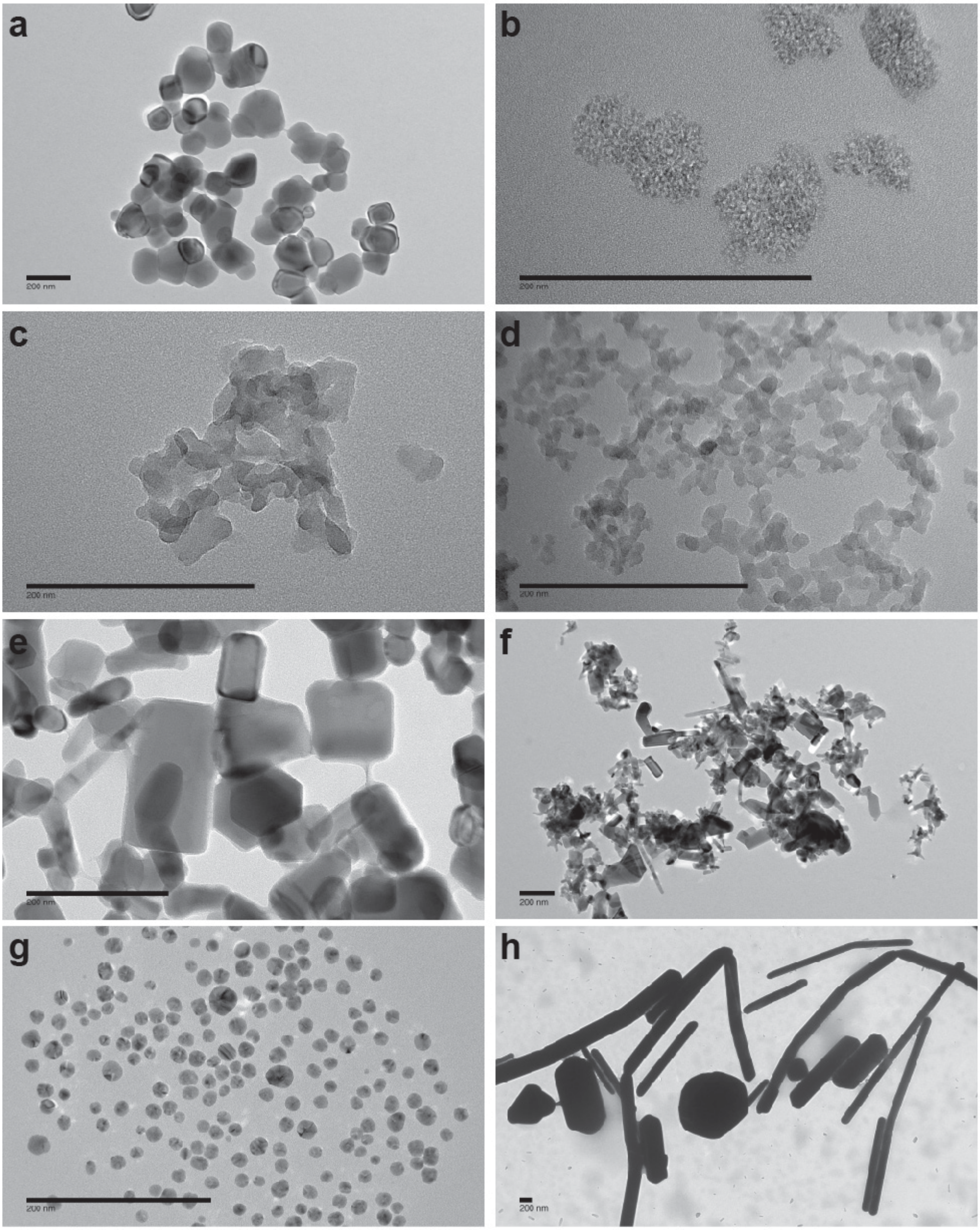

Figure S2: Transmission electron micrographs of NMs dispersed in 0.05 % w/v BSA-water. Morphology of **(a)** NM-100 (TiO_2), **(b)** NM-101 (TiO_2), **(c)** NM-200 (SiO_2), (**d**) NM-203 (SiO_2), (**e**) NM-110 (ZnO), (**f**) NM-111 (ZnO), (**g**) NM-300K (Ag), and (**h**) NM-302 (Ag-rods) (scale bar: 200 nm).

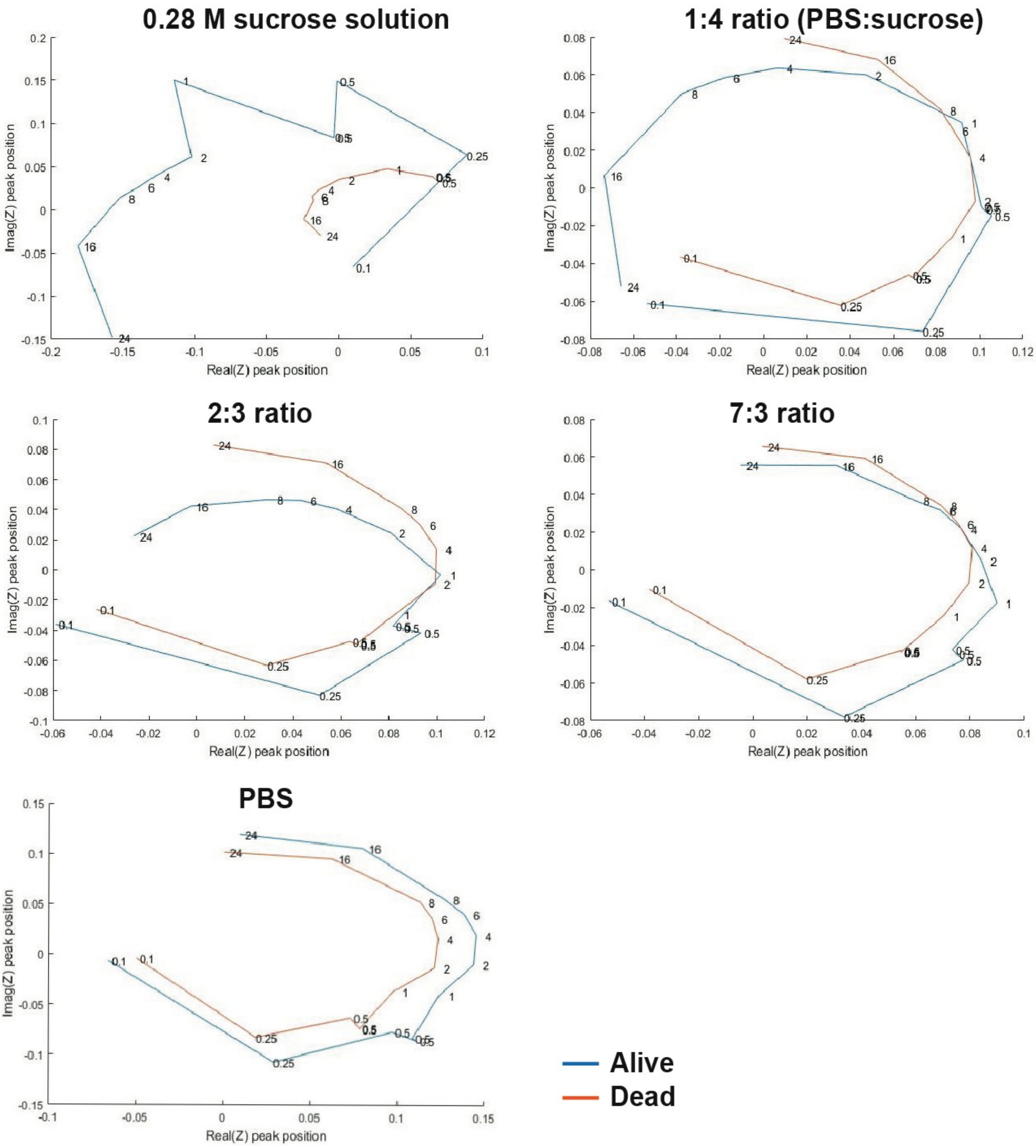

Figure S3: Impedance measurements of U937 cells using different sucrose-buffer concentrations and various frequencies. Imaginary (y-axis) and real part of impedance (x-axis) for the peak position (Gaussian approximation centre) were used to determine the sucrose-buffer concentration allowing an optimal differentiation of viable and necrotic cells for a given frequency. Buffer concentrations with ratio 2:3 (PBS : 0.28 M sucrose solution) and higher allows a separation of the two populations with increasing separation in the peak position. At 100% sucrose solution, artefacts appeared.

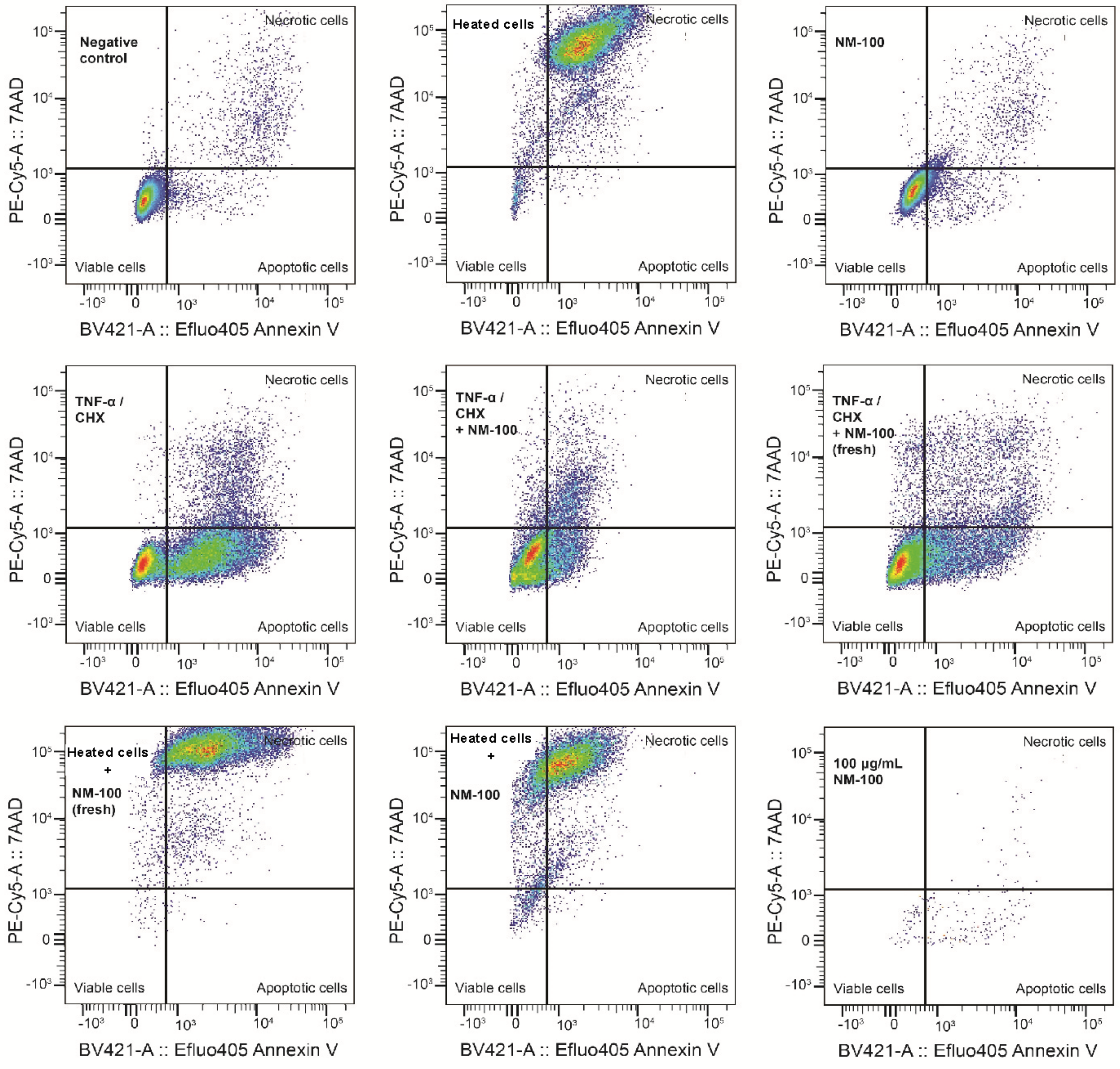

Figure S4: Representative flow cytometry dot plots for Annexin-V eFluor450 and 7-AAD staining.

To check for NM-induced interferences, 100 µg/mL NM-100 TiO2 particles were added prior to treatment of U937 cells with TNF-α / CHX (positive control for apoptosis) and to heating cells at 70°C for 30 min (positive control for necrosis). Annexin-V eFluor 450 and 7-AAD staining were used to detect apoptotic and necrotic cells. The addition of NM-100 prior to TNF- α /CHX treated cells resulted in an overestimation of viable (Annexin V- / 7-AAD-) cells as compared to cells treated only with TNF- α / CHX. The Annexin V- population increased when NM-100 were added prior to cell heating.

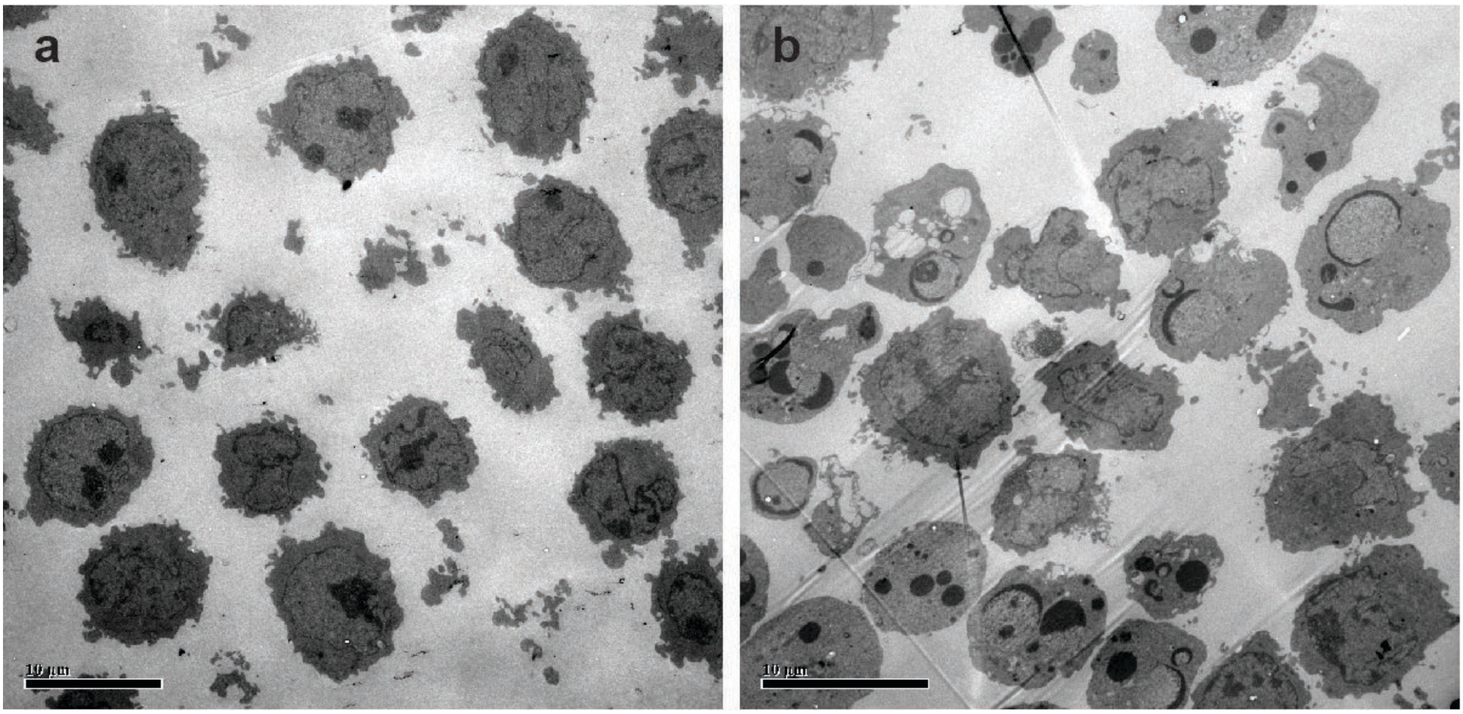

Figure S5: Transmission electron micrographs of U937 cells. (**a**) Viable (untreated) cells and (**b**) after TNF-α / CHX treatment. Scale bar 10 µm. (TEM image by CI Vamanu, published with permission).
